# Highly Sensitive Magnetic-Nanoparticle-Based Immunochromatography Assay for Rapid Detection of Amantadine in Chicken and Eggs

**DOI:** 10.3390/bios14010023

**Published:** 2023-12-30

**Authors:** Huaming Li, Yanrong Lu, Linwei Zhang, Liangni Qin, Hao Wen, Xiaohui Fan, Dapeng Peng

**Affiliations:** 1 State Key Laboratory of Agricultural Microbiology, National Reference Laboratory of Veterinary Drug Residues (HZAU) and MOA Key Laboratory for Detection of Veterinary Drug Residues, Huazhong Agricultural University, Wuhan 430070, China; lihm9915@163.com (H.L.); luli20100909@163.com (Y.L.); zhanglinwei9910@163.com (L.Z.); qinliangni123123@163.com (L.Q.); w793653717@163.com (H.W.); 2 Wuhan Shangcheng Biotechnology Co., Ltd., Wuhan 430070, China; 3 Shenzhen Institute of Nutrition and Health, Huazhong Agricultural University, Shenzhen 518000, China; 4 Shenzhen Branch, Guangdong Laboratory for Lingnan Modern Agriculture, Agricultural Genomics Institute at Shenzhen, Chinese Academy of Agricultural Sciences, Shenzhen 518000, China

**Keywords:** amantadine, residue detection, magnetic nanoparticles, immunochromatography, chicken, eggs

## Abstract

Amantadine (AMD) is an antiviral drug that is prohibited for use in livestock and poultry. In this study, carboxyl-modified magnetic nanoparticles (MNPs) were synthesized using the solvothermal method in one step with harmless and inexpensive regents, and they were used to label monoclonal antibodies (mAbs) of AMD in microwells with electrostatic adsorption. Then, a magnetic immunochromatography assay (MICA) method was successfully established. Under optimal conditions, the MICA showed a good performance, with a linear range of 0.2~10.0 µg/L. The limit of detection (LOD) was 0.068 µg/L with the instrument, and the visual LOD (vLOD) was 0.5 µg/L. There was no cross-reaction with rimantadine and ribavirin. The vLOD in real samples was 1.0 µg/kg. The developed MICA has the advantages of convenience, speed, and sensitivity, which make it suitable for the on-site rapid detection of AMD residues in chicken tissues and eggs.

## 1. Introduction

Amantadine (AMD) is a synthetic drug for the treatment of human influenza A virus disease and Parkinson’s disease [1]. Chronic exposure or consumption of AMD has been reported to damage the respiratory [2], reproductive [3], and nervous systems [4] in the human body. AMD has the characteristics of passing through the blood–brain barrier and excreting in breast milk, which leads to fetal malformation after consumption by pregnant women [5]. Extensive use of AMD can make people feel anxious, fatigued, and insomniac [6]. AMD can cause harm to human health after enrichment in the food chain [7], and residues in the environment increase the possibility of influenza virus mutation and reduce the effectiveness of disease treatment [8]. Therefore, the use of AMD in livestock and poultry breeding has been banned in China, the United States, and other countries. However, the announcement of notices (no. 37 2019, no. 12 2020, and no. 30 2020) by the State Administration for Market Regulation showed that AMD levels were still above limits in chicken and eggs sold in some supermarkets and farmers’ markets. Therefore, it is necessary to strengthen the residue detection of AMD residues in food.

The main detection methods for AMD are instrument methods, such as gas chromatography (GC) [9] and high-performance liquid chromatography–tandem mass spectrometry (HPLC-MS/MS) [10]. These instrument detection methods have a wide application range and high sensitivity, but they are not suitable for rapid detection in the field due to complex steps and professional operation. Therefore, it is necessary to develop simpler and more rapid methods for AMD residue detection. Immunological analysis based on antigen–antibody specific binding has been widely used for the detection of small-molecule compounds due to its high sensitivity, high specificity, and easy operation [11]. Commonly used immunological assays are enzyme-linked immunosorbent assay (ELISA) and immunochromatography assay (ICA). ELISA methods [12] have a long competition time and require multiple washes and the addition of chromogenic agents. ICA has the advantages of a short detection time, simple operation, and visual results, making it more suitable for on-site rapid detection. However, amantadine is a banned drug in animal tissues, and the sensitivity of existing ICA methods needs to be improved.

Magnetic nanoparticles have stable magnetic properties, biocompatibility, and rich chemical properties, which have been used for diverse applications including magnetic biosensing (diagnostics), magnetic imaging, magnetic separation, drug and gene delivery, hyperthermia therapy, etc. [13]. The method of combining magnetic nanoparticles with ICA is called MICA, which exhibits strong magnetic signal penetration and lower biological background interference [14] than ELISA [15], making it more convenient for detection at point-of-care. Recently, MICA has been used for the determination of toxins [16], pathogens [17], viruses [18], and biomarkers [19], but its application for amantadine residue detection has not been reported. In addition, many of the currently reported methods for the synthesis of MNPs, such as chemical coprecipitation [20], thermal decomposition [21], and microemulsion [22], are complex and expensive, hindering the commercial development of MICA.

In this work, carboxyl-modified MNPs were synthesized using a one-step solvothermal method at a low cost and then coupled with mAbs against AMD to prepare magnetic nanoimmunoprobes (MNPs–mAbs). On this basis, a highly sensitive MICA for the rapid detection of AMD residues in chicken tissues and eggs was established for the first time. The LOD of this method was 0.068 μg/L when the magnetic signal was collected with the instrument and 0.5 μg/L when visually observed. In order to facilitate rapid detection in the field, this study only made visual judgments on the detection limit of samples, which was 1.0 μg/kg. Compared with the existing strip methods, this method has the advantages of high sensitivity, high specificity, and a short detection time, and is more suitable for on-site detection.

## 2. Material and Methods

### 2.1. Materials

Standard analytes of 1-amantadine (98%), rimantadine hydrochloride (100%), and ribavirin (98%) were bought from Bailingwei Technology Co., Ltd. (Beijing, China). The coating antigen AMD-BSA (4.5 mg/mL) and the mAb of AMD (2.6 mg/mL; IC_50_ = 1 µg/L) were bought from Ditengmin Biotechnology Co., Ltd. (Wuxi, China). Goat anti-mouse immunoglobulins (IgG, 5.4 mg/mL), horseradish-peroxidase-labeled goat anti-mouse immunoglobulins (HRP-IgG, 0.8 mg/mL), and 3,3′,5,5′-tetramethylbenzidine (TMB) were obtained from Shangcheng Biotechnology Co., Ltd. (Wuhan, China). Ethylene glycol and ferric chloride hexahydrate were acquired from Macklin Biotechnology Co., Ltd. (Shanghai, China). Ethylenediamine-N-propylsilane was bought from JADELLchem Technology Co., Ltd. (Shanghai, China). Anhydrous sodium acetate, acetonitrile, methanol, ether, trisodium citrate dihydrate, Tween-20, and polyvinylpyrrolidone (PVP) were purchased from Sinopharm Chemical Reagent Co., Ltd. (Shanghai, China). The nitrocellulose membrane (NC membrane), sample pad, and absorbent paper were bought from Jieyi Biotechnology Co., Ltd. (Shanghai, China). Microwells were obtained from Guosheng Biotechnology Co., Ltd. (GuangdongShenzhen, China). Commercial MNPs of 30, 100, and 200 nm were purchased from ZhongkeLeiming Technology Co., Ltd. (Beijing, China). The dispenser system BioDot-XYZ3210 and high-speed CNC cutting machine CM3020 were purchased from Jiening Biotechnology Co., Ltd. (Shanghai, China). The magnetic assay reader (MAR™) was bought from MagnaBioSciences (San Diego, CA, USA). The SEM testing/field-emission scanning electron microscopy Zeiss Supra55 was obtained from Carl Zeiss AG (Oberkochen, Baden-Württemberg, Germany). The nanoparticle size/zeta potential analyzer Zetasizer Nano ZS was acquired from Malvern Instrument Ltd. (Great Malvern, UK). The VERTEX 70 Fourier-transform infrared spectrometer was acquired from Bruker Corporation (Karlsruhe, Baden-Württemberg, Germany).

### 2.2. Synthesis and Characterization of MNPs

MNPs were prepared using the solvothermal method reported with slight modifications [23]. Ethylene glycol (20 mL), FeCl_3_∙6H_2_O (0.70 g), and trisodium citrate dihydrate (0.25 g) were added to a 50 mL beaker and stirred in a magnetic stirrer for 80 min. After the solid was completely dissolved, sodium acetate (1.20 g) was added and agitated for 30 min. The mixture was poured into a Teflon-lined stainless-steel autoclave, sealed completely, and then placed into an oil bath at 200 °C for 8 h. After cooling at an ordinary temperature, the mixture was divided into beakers and washed three times with ethanol and deionized water. After magnetic separation, the product was placed in a vacuum-drying oven at 60 °C for 8 h. The brown product was the carboxyl-modified MNPs, which were diluted with deionized water to 10 mg/mL in a tube and stored at 4 °C for later application.

The magnetism, structure, size, surface potential, and constituent elements of the MNPs were characterized with an external magnetic field, scanning electron microscope, nanoparticle size/zeta potential analyzer, and Fourier-transform infrared spectrometer.

### 2.3. Preparation of MNP–mAb Microwells

MNPs (100 µg) and mAbs (5 µg) were added to 1 mL of deionized water and then mixed and reacted for 60 min at room temperature. The product was obtained after magnetic separation and washing with deionized water and confirmed using ELISA. Firstly, the coating antigen (AMD-BSA) was coated on microwells for 2 h at 37 °C. After washing 3 times with PBST (0.1 mol/L of PBS containing 0.1% Tween-20), MNPs and MNPs–mAbs were added (100 μL/well) separately and incubated for 30 min. After washing 3 times, the HRP-IgG (1:5000 dilution) was added (100 μL/well) and incubated for 30 min. After washing 3 times, TMB solution was added (150 μL/well) and incubated for 15 min. Finally, the color was compared to determine whether the mAbs conjugated to the MNPs.

MNP–mAb microwells were prepared for the reaction of AMD with the MNPs–mAbs. Single-factor screening was performed on the amount of mAb, coupling solution, coupling time, and blocking time in the above labeling process, and MNPs–mAbs were prepared under optimal conditions. Deionized water (1 mL) and a 10 mg/mL MNP (10 µL) solution were added to a tube, and the MNPs were washed twice with deionized water after mixing. After magnetic separation, they were resuspended in 1 mL of a coupling solution (deionized water; a 0.01 mol/L boric acid solution (pH 6.7); and 0.01 mol/L phosphate-buffered saline (PBS) (pH 7.4)). An appropriate amount of mAb (2 µg/3 µg/5 µg) was added to the mixture, and then it was mixed and reacted at room temperature for 15~60 min. A blocking solution (100 µL of 10% BSA) was added to the mixture, which was then placed in a rotator and allowed to react for 15~60 min at room temperature. The MNPs–mAbs were resuspended in 100 µL of a reconstituted solution (0.5% BSA) after magnetic separation and stored at 4 °C. A lyophilized solution (5% BSA, 2% sucrose, and 0.5% Tween-20) and MNPs–mAbs were mixed at 4:46 (*v*/*v*) and appended to the microwells (50 μL/well). Subsequently, all microwells were lyophilized and stored in a dry environment.

**Figure 1:**
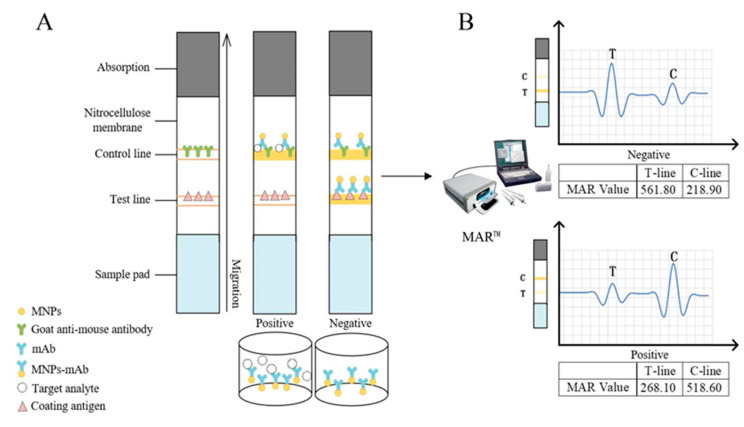
Schematic illustration of the competitive MICA. (**A**) MNPs-mAbs composite microwell reaction system and the structure of MICA; (**B**) The MAR™ detects the magnetic signals on the C&T line of the strip and expresses them as corresponding positions, amplitudes, and values.

The structure of the immunochromatographic strip is shown in Figure 1A. The NC membrane, the sample pad, and the absorbent paper were pasted on the PVC plate in turn, and the adjacent parts overlapped by 2 mm. After assembling, the strip was cut to 3 mm with CM3020. The MNP–mAb microwells and the desiccant were put into an aluminum foil bag and sealed for later use.

### 2.4. Assembly of MICA Strip and Optimization of MICA

The AMD-BSA and IgG were sprayed on the test line(T-line) and control line(C-line) of five different NC membranes (Boya A × C 100, Boya Cn 100, PALL VIV17025 50R, PALL VIV12025 100R, and PALL VIV9025 100R) with the BioDot XYZ3210 platform, and the chromatographic status of the MNPs–mAbs on different nitrocellulose (NC) membranes was observed to select the best.

The combination of the coating antigen AMD-BSA (0.05 mg/mL and 0.07 mg/mL) and goat anti-mouse immunoglobulins (IgG; 0.7 mg/mL and 1 mg/mL) was striped on the T-line and C-line at an interval of 5 mm. The test strips were assembled and inserted into the MNP–mAb microwells containing 100 μL of an AMD standard solution (0.5 μg/L and 2 μg/L) and a blank solution, respectively. Ten minutes later, they were detected using the magnetic assay reader (MAR™). The combination with the highest inhibition rate was selected as the optimal antigen AMD-BSA and IgG coating concentration.

The ability of the AMD in the tested sample to compete with the T-line coating antigen AMD-BSA of the test strip to bind MNPs–mAbs is expressed by the inhibition rate (Formula (1), I = T/C, where I_0_ is defined as T/C when the AMD concentration in the sample is 0 μg/L). The greater the inhibition rate, the more targets are contained in the tested sample.

(1) $$\mathrm{Inhibition}\;\mathrm{rate}=\left(1-\frac{I}{I_{0}}\right)\times \;100\%$$

### 2.5. Procedure of MICA

As shown in Figure 1B, the strip for detecting the concentration of the samples in the microwells was inserted into the MAR™, and the instrument was run to detect the magnetic signal intensities of the C-line and T-line. The magnetic test line traveling through the core produced a characteristic response, which was plotted as amplitude and position data. The amplitude and position data were reported as MAR values. MAR was a unitless number.

The MNP–mAb microwells and the test strips were taken out of the aluminum foil package. The sample solution (100 μL) was added into the MNP–mAb microwells, mixed well, and reacted. The test strip was inserted into the microwells for a period of time and taken out to detect the magnetic signals of the C-line and T-line. Single-factor screening was performed on the reaction and chromatography time under the above detection procedure, and the detection was carried out under optimal conditions.

The detection results were determined according to the principle of competitive immunochromatography. If the sample contains the target, the target will bind to the MNPs–mAbs, and the MNPs–mAbs will not be enriched by the T-line. The MAR value of the T-line will be significantly lower than that of the C-line (T/C < 1.0), indicating the result is positive. On the contrary, if the sample does not contain the target, the MNPs–mAbs will be captured by the C-line and T-line, and the MAR value of the C-line will be consistent with or lower than that of the T-line (T/C ≥ 1.0), indicating the result is negative. When the MAR value of the C-line does not exist, the detection result is invalid.

### 2.6. Sample Preparation

Samples (chicken and eggs) were purchased at the local market and identified as blank samples without AMD using LC-MS/MS performed by Jia [24]. The skins and bones of the chickens and the shells of the eggs were removed, and then the samples were homogenized. Homogeneous samples (2 g) were placed into a 50 mL test tube and an appropriate concentration of AMD was added. The effects of the sample treatments with PBS, ethyl acetate, acetonitrile + NaCl, acetonitrile–ethyl acetate (1:1) + NaCl, acetonitrile with 1% acetic acid + NaCl, and 1% hydrochloric acid–acetonitrile + 0.50 g of NaCl were compared in this study, and the specific protocols are shown in Table S1. After the addition of each extraction agent, the tube was violently shaken for 5 min and centrifuged at 4000× *g* for 3 min. The supernatant (2 mL) was added to a 10 mL centrifuge tube and desiccated with a sample concentrator at 65 °C. Redissolved solvents were added into the 10 mL tube and swirled for 1 min. After centrifugation at 4000× *g* for 1 min, the subnatant, called the sample solution, was detected via MICA.

### 2.7. Performance of MICA

Under optimal conditions, different concentrations of the AMD standard solution (0, 0.2, 0.5, 1, 5, and 10 µg/L) were detected using MICA to establish a standard curve and evaluate the sensitivity of the MICA. The same concentration of AMD, rimantadine, and a ribavirin standard solution (100 µg/L) were detected using MICA while the blank control group test was performed. The specificity of the MICA was evaluated by the difference between the above results.

The final specifications of the spiked samples were 0, 0.2, 0.5, 1.0, 5.0, and 10.0 μg/kg of AMD added to 2 g of blank chicken samples. After the sample preparation, the sample solution was tested using the MICA method.

### 2.8. Comparison of MICA and LC-MS/MS Analysis

Four white-feathered chickens were acquired from the MOA Laboratory for the Risk Assessment of the Quality and Safety of Livestock and Poultry Products (Wuhan, China). All animal experiments in this study were approved by the Animal Ethics Committee and conducted according to the guidelines of the Animal Experimentation Center of Huazhong Agricultural University. The ethical number was HZAUMO-2021-0179. Housing and slaughter were conducted according to the ethical and animal care guidelines. The chickens were fed a diet without AMD. After 7 d, they were weighed and orally administered AMD at 0, 2.5, 5.0, and 10.0 mg/kg, respectively. Five hours later, the animals were slaughtered after CO_2_ anesthesia, immediately followed by the dissection of the chickens. The chicken tissues (chicken and liver) were homogenized and divided evenly into several parts.

The sample (2 g) was placed into a 50 mL tube, and 10 mL of the extraction agent (1% acetonitrile acetate) added. After shaking violently for 5 min, the mixture was centrifuged at 4000× *g* for 5 min. The supernatant was transferred into another 50 mL tube, and anhydrous sodium sulfate (3 g) and N-hexane (10 mL) were added. The tube was vortexed for 1 min, centrifuged at 4000× *g* for 5 min, and the N-hexane layer discarded. The remaining solution was transferred to a 10 mL tube and dried in nitrogen at 40 °C. The residue was dissolved with 0.5 mL of a 50% acetonitrile aqueous solution, and 50 mg of ethylenediamine-N-propylsilane was added, and then the mixture was vortexed for 30 s and centrifuged at 8000× *g* for 5 min. The supernatant was filtered with a 0.22 μm membrane and taken for LC-MS/MS determination. The LC-MS/MS method referred to the study by Tsuruoka et al. [25]. All samples were detected via MICA at the same time.

## 3. Results and Discussion

### 3.1. Commercial MNPs

**Figure 2:**
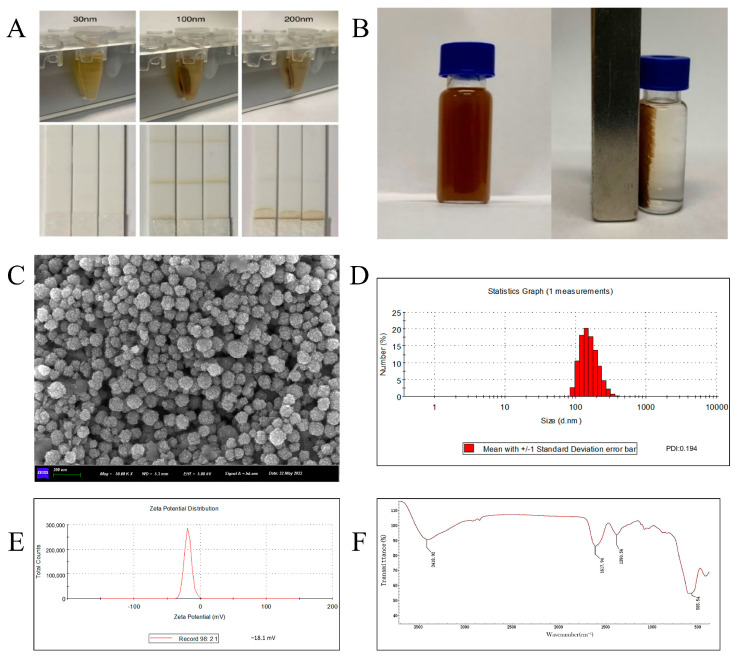
Characterization of MNPs synthesized in this study. (**A**) The imparities of MNPs with different sizes in magnetic separation and chromatographic status for MICA; (**B**) the magnetic response of MNPs under the outside magnetic field; (**C**) the SEM image of MNPs; (**D**) the hydrate particle size of MNPs; (**E**) the zeta potential of MNPs; and (**F**) the infrared spectrum of MNPs.

The particle size of nanoparticles has a significant impact on their performance [26]. The magnetic nanoimmunoprobes (MNPs–mAbs) were prepared by coupling AMD monoclonal antibodies with commercial MNPs at 30, 100, and 200 nm to select suitable MNPs for MICA. It was found that there were significant imparities in the effects of MNPs with different particle sizes used in immunochromatographic strips. The 30 nm MNPs could not be magnetically separated due to their low magnetism and could not be completely precipitated via high-speed centrifugation, so there was a large loss of MNPs in the process of marking on the NC membrane, which resulted in the absence of color on the C-line and T-line. The magnetic separation effect of the 100 nm MNPs was good. There was no obvious agglomeration during labeling. The color of the C-line and T-line was obvious during the detection of negative samples. However, the magnetic separation effect of the 200 nm MNPs was worse, and there was a reasonable accumulation of MNPs–mAbs at the connection place between the NC membrane and the sample pad during the detection of negative samples, while the C-line and T-line had no color. In conclusion, 100 nm was the optimal size of MNPs used in MICA (Figure 2A).

### 3.2. Synthesis and Characterization of MNPs

There are many synthetic methods for MNPs with different advantages and disadvantages. In order to ensure that MNPs used in MICA meet the characteristics of uniform particle size and good dispersion, 100 nm MNPs were synthesized using a solvothermal method.

As shown in Figure 2B, MNPs were uniformly dispersed in deionized water when no external magnetic field was applied. After adding the magnetic field, the MNPs were adsorbed to one side, and the rest of the liquid was transparent, which indicated that the MNPs could be well separated by means of a magnetic gradient field. The surface morphology and size of the MNPs were inspected using scanning electron microscopy. The MNPs were spherical in shape, with an average size of 100 nm, which is shown in Figure 2C. The state of the MNPs in water was analyzed using a nanoparticle size/zeta analyzer. The PDI index in water was 0.194, and the dispersion range of the particle size was mainly concentrated at 100~200 nm, indicating that they can be dispersed in an aqueous solution without agglomeration (Figure 2D). In addition, the surface potential of the MNPs in water was −18.1 mV, which was consistent with the potential of carboxyl groups in water (Figure 2E). The surface groups of MNPs were determined with an infrared spectrometer. The spectrogram in Figure 2F contains all the peaks of the MNPs. The absorption characteristic peaks of Fe-O and -OH appeared at 585.84 cm^−1^ and 3418.92 cm^−1^, respectively. The absorption characteristic peaks of the carboxyl group appeared at 1617.94 cm^−1^ and 1390.56 cm^−1^. This indicates that the carboxyl groups were successfully connected to the surfaces of the MNPs. The magnetic nanoparticles synthesized in this study with negatively charged carboxyl groups on their surfaces are similar to colloidal gold and can bind to proteins via electrostatic adsorption and hydrogen bonding [27].

In this work, fewer reagents and materials were used, the chemical synthesis conditions were safe and efficient, and the obtained MNPs met the requirements of the MICA method.

### 3.3. Preparation of MNP–mAb Microwells

At present, most MNPs–mAbs are prepared by activating the surface carboxyl group of the MNPs to bind with the antibodies via chemical bonds [28,29]. This study discovered that the surfaces of the MNPs had negative charges, and the electrostatic adsorption occurred when the MNPs and mAbs were incubated together. The successful conjugation of MNPs–mAbs was identified using ELISA. If mAbs are successfully conjugated to the magnetic nanoparticles, the formed MNP–mAb complexes can recognize and bind the coated antigen AMD-BSA in ELISA, and after washing, HRP-IgG incubation, and TMB color development, a blue solution is formed. Conversely, MNPs unconjugated to mAbs are washed and not visualized. The results shown in Figure S1 indicate that MNPs and mAbs were conjugated successfully.

MNPs and mAbs were added to microwells to form MNPs–mAbs, which specifically bound to AMD to form MNPs–mAbs–AMD when the samples contained AMD. In the strip chromatography, the MNPs–mAbs–AMD were no longer captured by the coating antigen, resulting in weak magnetic signals at the T-line. Furthermore, the electrostatic adsorption between the MNPs and antibodies in the microwells simplified the steps, reduced the operation time, and required fewer reagents than chemical coupling.

**Figure 3:**
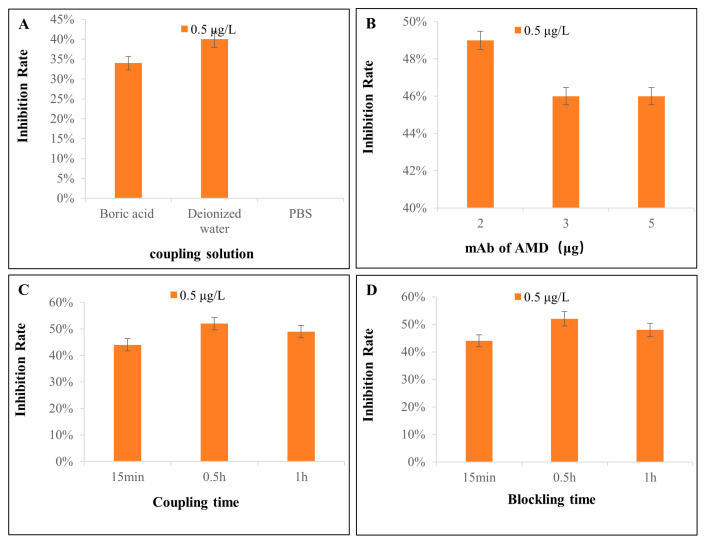
The inhibition rate of MICA in AMD standard solution (0.5 μg/L) under various optimization conditions. (**A**) The inhibition rate of MNPs labeled with mAbs in different coupling solutions; (**B**) the inhibition rate of different mAb amounts labeled with 100 µg MNPs; (**C**) the inhibition rate of coupling MNPs with mAbs for different times; and (**D**) the inhibition rate of blocking redundant sites on MNPs for different times. Error bars show standard deviations (*n* = 3).

During the experiment, the coupling conditions of MNPs with mAbs of AMD were screened. It was found that the proportion of MNPs and mAb, coupling solution, and coupling and blocking time had a great influence on the specific recognition ability of the MNPs–mAbs. Especially, the dispersibility and sensitivity of the MNPs–mAbs varied in different solution systems. The results in Figure 3A indicate that MICA had the best inhibition rate in deionized water.

Insufficient antibodies will lead to too many empty binding sites in MNPs. If the blocking is not complete, this will lead to an increase in non-specific binding, and the strip will be prone to false negatives. Even if the redundant sites are completely blocked with the blocking solution, the number of antibodies on the MNPs will be too small, which will make it easy for them to be saturated by the target antigen in the sample, resulting in an increase in false positives. Too many antibodies will not only reduce the sensitivity but also waste raw materials. The results in Figure 3B show that the best proportion of MNPs to antibodies was 100 µg:2 µg.

The antibodies can occupy most sites on the MNPs to saturate them for the appropriate coupling time. In addition, the screening of the blocking time can determine the minimum time to completely block the redundant vacancies on the MNPs. An excessive blocking time may increase the risk of MNP agglomeration. The optimization results of the coupling time and blocking time are depicted in Figure 3C,D. The results show that the binding ability of the MNPs–mAbs to the antigen was the best when the coupling and blocking times were both 30 min.

### 3.4. Optimization of MICA

**Figure 4:**
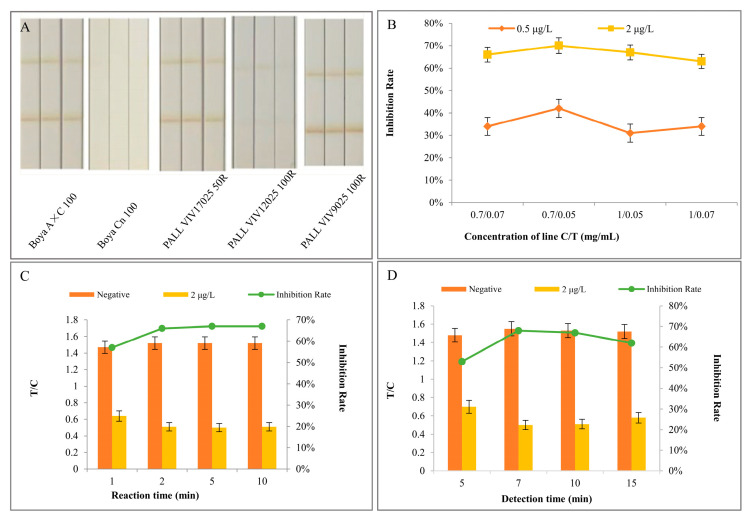
Optimization of MICA. (**A**) The chromatographic performance of MNPs–mAbs on various NC membranes; (**B**) the inhibition rate of MICA at different concentrations of the AMD-BSA and IgG; (**C**) the inhibition rate of MICA for various reaction times of MNPs–mAbs with the target antigen; and (**D**) the inhibition rate of MICA for various detection times of the strip. Error bars show standard deviations (*n* = 3).

In this experiment, the factors affecting the sensitivity of MICA were optimized. It was found that the chromatographic performance of MNPs–mAbs on different NC membranes varied with the material. The results of the NC membrane screening shown in Figure 4A indicate that the MNPs–mAbs can be smoothly chromatographed on three models of NC membranes: Boya A × C100, PALL VIV17025 50RPALL, and VIV9025 100R. The NC membrane background was clean, and there was no MNP–mAb accumulation at the connection with the sample pad. Comparing the three, the C-line and T-line on the PALL VIV9025 100R were more intensely visualized, indicating that MNPs–mAbs were more completely chromatographed on this membrane, and, therefore, it was identified as the optimal NC membrane for use in MICA.

A combination of the IgG (0.7 mg/mL and 1 mg/mL) and the antigen AMD-BSA (0.05 mg/mL and 0.07 mg/mL) was sprayed on the C-line and T-line of the strips. Then, the strips were inserted into MNP–mAb microwells containing 100 μL of the AMD standard solution (0.5 μg/L and 2 μg/L) and a blank solution. The inhibition results in Figure 4B indicate that the optimal concentration of the IgG (C-line) was 0.7 mg/mL, and the concentration of the antigen (T-line) was 0.05 mg/mL.

In addition, the reaction time determines whether the MNPs–mAbs react completely with the antigen in the test solution. MNPs–mAbs were reacted with 100 μL of the AMD standard solution (2 μg/L) for 1, 2, 5, and 10 min, respectively, and detected with MICA, using PBS as the negative control. The results in Figure 4C show that the optimal reaction time between the sample solution and MNPs–mAbs was 2 min.

A reasonable detection time can avoid the influence of the “post-display” phenomenon on the test results and prevent it from reducing the sensitivity of the test strip. As shown in Figure 4D, the ideal chromatography detection time of strips was 7 min.

### 3.5. Sample Preparation

Sample pretreatment is an important part of MICA. In this work, nine sample preparation methods were compared for chicken and egg. The difference between positive and negative values was taken as an indicator, and the results are shown in Table S2. The composition of animal samples was complex, and there were strong false positives using PBS extraction. AMD cannot be extracted with ethyl acetate, while acetonitrile can. Hydrochloric acid added to egg samples can denature the protein, thus strengthening the extraction effect. Moreover, the addition of a high concentration of sodium chloride increases the separation of organic and aqueous phases. It turned out best when 2 g of chicken tissue was extracted with 5 mL of acetonitrile + 0.5 g of NaCl and redissolved with 300 μL of PBST + 1 mL of N-hexane. Similarly, 2 g of egg sample was extracted with 5 mL of 1% hydrochloric acid–acetonitrile + 1.0 g of NaCl and redissolved with 400 μL of PBST + 1 mL of N-hexane.

### 3.6. Performance of MICA

**Figure 5:**
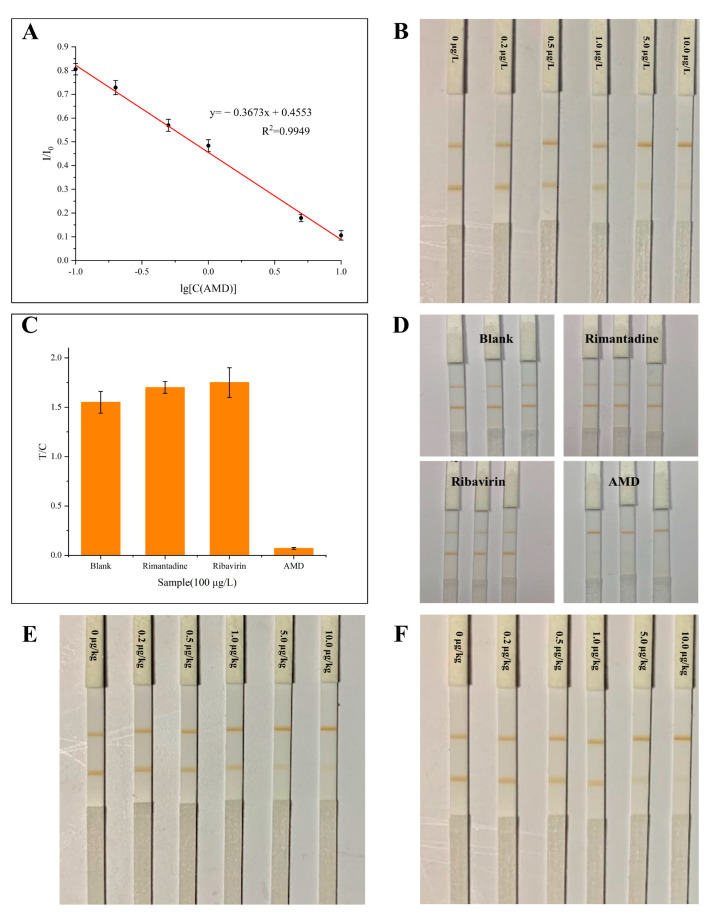
The performance of MICA. (**A**) Standard curve of MICA; (**B**) colorimetric test results of standard solutions; (**C**) specificity of MICA; (**D**) colorimetric test results of specificity; (**E**) colorimetric test results of different concentrations in eggs; and (**F**) colorimetric test results of different concentrations in chicken. Error bars show standard deviations (*n* = 3).

The AMD standard solution was diluted to 0.2, 0.5, 1, 5, and 10 µg/L and tested with MICA under optimal conditions, and the magnetic signals were analyzed to obtain a standard curve, as shown in Figure 5A. The relationship between I/I_0_ (defined as y) and the lg[C(AMD)] (defined as x) was linear in the range of 0.2~10.0 µg/L. The standard curve equation was y = − 0.3673x + 0.4553, R^2^ = 0.9949. Twenty 0 µg/L standard solutions were detected with MICA. The limit of detection (LOD, where LOD = mean of blank + 3 × standard deviation) was 0.068 µg/L, and the limit of quantitation (LOQ, where LOQ = mean of blank + 10 × standard deviation) was 0.126 µg/L. The strips of MICA detecting the AMD standard solution are shown in Figure 5B, and the visual LOD (vLOD), defined as the minimum AMD concentration where the color of the T-line was lighter than the C-line, was 0.5 µg/L.

The standard solution (100 µg/L) of AMD, rimantadine, ribavirin, and the blank solution were detected using MICA. The specificity results are shown in Figure 5C,D. The value of the T/C of the AMD solution was less than 0.2, while those of rimantadine, ribavirin, and the blank solution were larger than 1.5, indicating that the MICA had good specificity.

The main application scenario for ICA detection is rapid qualitative detection in the field; therefore, the sample sensitivity of MICA magnetic signals was not tested in this study, only the visual LOD in eggs and chicken was tested. The results shown in Figure 5E,F depict that the vLOD values of eggs and chicken were both 1.0 μg/kg using MICA in this method.

### 3.7. Validation of MICA

Actual samples including negative and positive samples were detected. The colorimetric results detected with MICA are shown in Figure S2. The mass spectrometry of the AMD standard solution and real sample results detected with LC-MS/MS are shown in Figure S3. The results show that the determination results of the AMD residues in actual samples with MICA were consistent with those using LC-MS/MS (Table S3). All these results indicate that the MICA in this work is reliable for the rapid on-site detection of AMD.

### 3.8. Comparison with Reported Methods

Several reported methods and the MICA established in this work are shown in Table 1. MICA has the advantages of a higher sensitivity, shorter time required, and simpler operation than reported methods. In addition, the magnetic nanoparticles used in this study have the characteristics of stable magnetism and low biological background interference, which have great advantages in sample processing and high application value in rapid detection in the field.

## 4. Conclusions

In this study, MNPs were synthesized in one step with a harmless and inexpensive reagent. The MNPs were used to couple with antibodies and a highly sensitive MICA was established and applied for the rapid detection of AMD residues in chicken tissues and eggs for the first time. The LOD of the MICA was 0.068 μg/L with the instrument and 0.5 μg/L with visual colorimetry, and the vLOD in real samples was 1.0 μg/kg, which is superior to existing ICA methods. This method required only 9 min for incubation and detection, which is quicker than reported methods, indicating this method has great advantages for rapid detection in the field. Eight actual samples were analyzed using both MICA and LC-MS/MS, and the results imply that the MICA in this work is reliable.

## Figures and Tables

**Table 1:** Comparison between reported methods and this work.

Method	Property	vLOD (μg/kg)	LOD (μg/kg)	Linear Range (μg/kg)	Total Time (min)
CGICA [12]	Qualitative	5.0	0.62	-	5
CGICA [30]	Qualitative	10.0	-	-	10
CGICA [31]	Quantitative	-	1.8	2.5~25.0	12
bFQICA [32]	Qualitative/quantitative	-	0.62	1.07~10.33	18
TRFICA [32]	Quantitative	-	0.29	0.37~19.46	11
SA-QDs-ICTS [33]	Quantitative	-	0.18	0.23~1.02	15
AuNCs-FQICTS [34]	Qualitative/quantitative	2.5	0.45	0.5~25	23
LFIA [35]	Qualitative/quantitative	1.0	0.5	0.5~10.0	15
MICA (this work)	Qualitative/quantitative	1.0	0.068	0.2~10.0	9

## Data Availability

The data presented in this study are available on request from the corresponding author.

## Supplementary Materials

The following supporting information can be downloaded at: https://www.mdpi.com/article/10.3390/bios14010023/s1, Figure S1: MNPs-mAbs confirmed by ELISA; Figure S2: Colorimetric results of real samples detected by MICA; Figure S3: Mass spectrum of AMD in standard solution(5.0 μg/L) and actual samples; Table S1: Methods of sample preparation; Table S2: Difference between positive and negative values of pretreatment methods; Table S3: Comparison between MICA and LC-MS/MS.
