# Supplementary material for: Highly Sensitive Magnetic-Nanoparticle-Based Immunochromatography Assay for Rapid Detection of Amantadine in Chicken and Eggs
Source: Biosensors (Basel). 2023 Dec 30;14(1):23. doi: 10.3390/bios14010023 (PMC10813809; doi:10.3390/bios14010023)
Supplement: Supplementary file 1 [file biosensors-14-00023-s001.zip › biosensors-2738984-supplementary.pdf]

Supplementary materials:

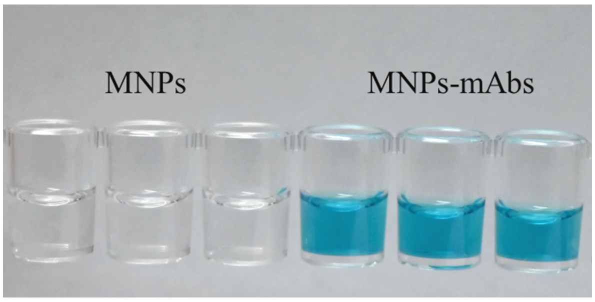

Figure S1: MNPs-mAbs confirmed by ELISA

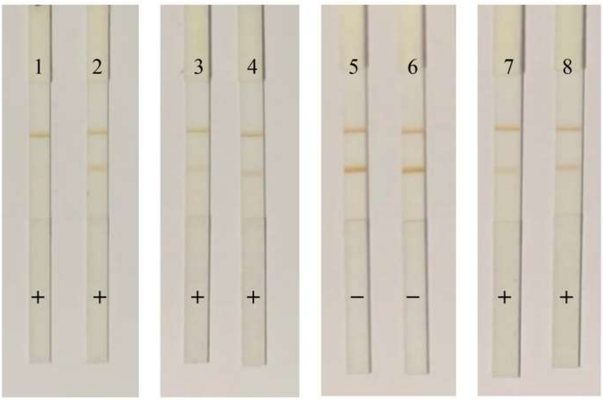

Figure S2: Colorimetric results of real samples detected by MICA.

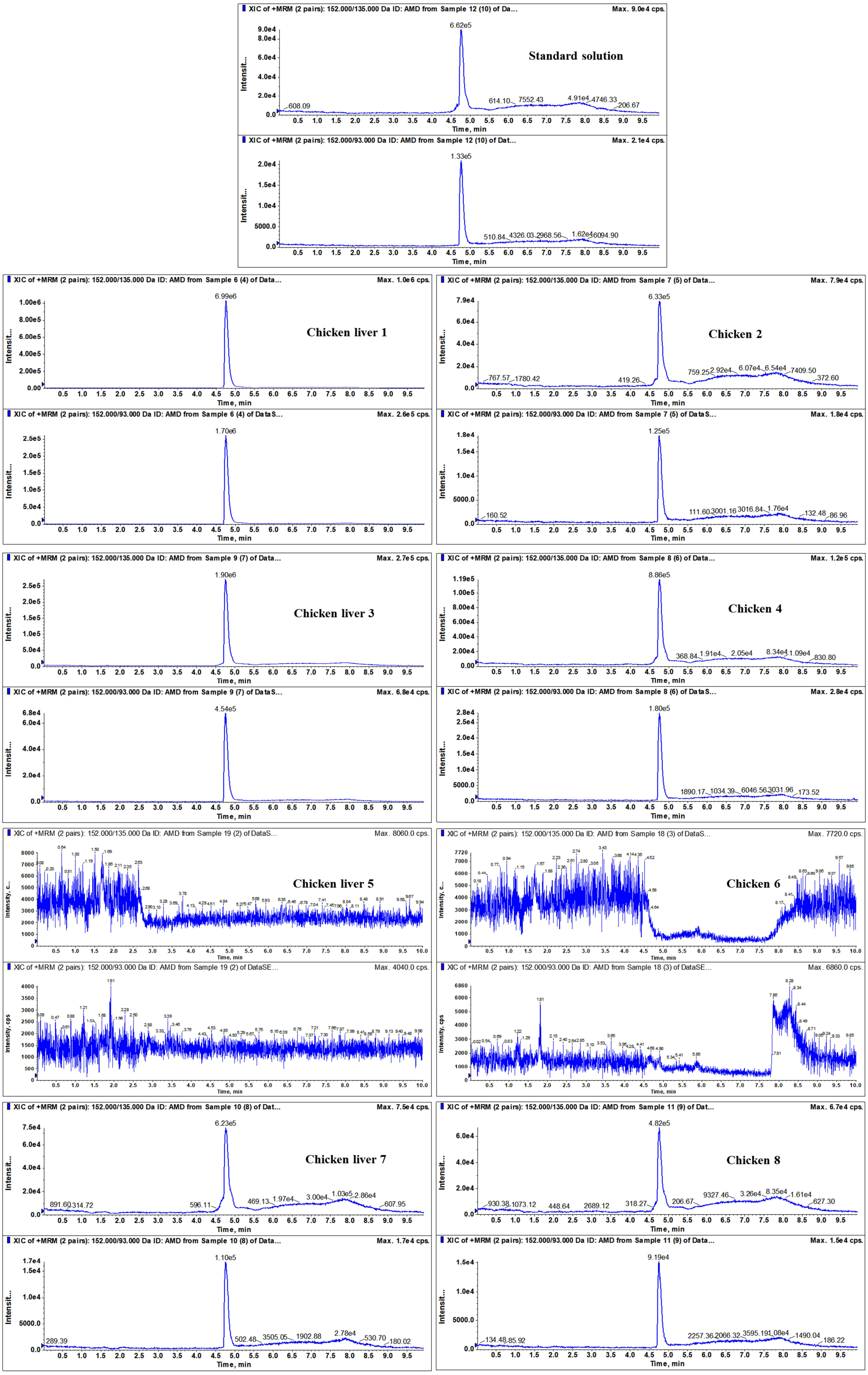

Figure S3: Mass spectrum of AMD in standard solution(5.0 μg/L) and actual samples

Table S1: Methods of sample preparation

Method	Sample	Weight (±0.05g)	Extractant	Redissolve solvents
1	chicken	2	5 mL PBS（pH7.4）	−
2	chicken	2	5 mL Ethyl acetate	300 μL PBST+ 1 mLN-hexane
3	chicken	2	5 mL Acetonitrile+ 0.5 g NaCl	300 μL PBST+ 1 mLN-hexane
4	chicken	2	5 Ml Acetonitrile-Ethyl acetate(1:1)+0.5 g NaCl	300 μL PBST+ 1 mLN-hexane
5	egg	2	5 mL Acetonitrile+ 0.5 g NaCl	400 μL PBST+ 1 mLN-hexane
6	egg	2	5 mL Acetonitrile with 1% acetic acid +0.5 g NaCl	400 μL PBST+ 1 mLN-hexane
7	egg	2	5 mL Acetonitrile with 1% acetic acid +1.0 g NaCl	400 μL PBST+ 1 mLN-hexane
8	egg	2	5 mL 1% Hydrochloric acid-acetonitrile+0.5 g NaCl	400 μL PBST+ 1 mLN-hexane
9	egg	2	5 mL 1%Hydrochloric acid-acetonitrile+1.0 g NaCl	400 μL PBST+ 1 mLN-hexane

Table S2: Difference between positive and negative values of pretreatment methods

Method	Sample	Difference	Method	Sample	Difference
1	chicken	−	5	egg	++
2	chicken	−	6	egg	−
3	chicken	++++	7	egg	−
4	chicken	++	8	egg	+++
			9	egg	++++

−: no difference; +: the larger the number, the larger difference.

Table S3: Comparison between MICA and LC-MS/MS

Sample		MICA	LC-MS / MS	
chicken liver	1	+	+	
chicken	2	+	+	
chicken liver	3	+	+	
chicken	4	+	+	+: positive
chicken liver	5	−	−	−: negative
chicken	6	−	−	
chicken liver	7	+	+	
chicken	8	+	+	
